# Regulatory cytokines modulate early isotype-specific response associated with COVID-19 survival

**DOI:** 10.3389/fimmu.2025.1543626

**Published:** 2025-04-24

**Authors:** Sebastián Castro-Trujillo, Juanita Castro-Meneses, María Clemencia Rojas, Marcela Castro-Amaya, Giovani Lastra, Carlos F. Narváez

**Affiliations:** ^1^ División de Inmunología, Programa de Medicina, Facultad de Ciencias de la Salud, Universidad Surcolombiana, Neiva, Huila, Colombia; ^2^ Programa de Biología Aplicada, Facultad de Ciencias Exactas y Naturales, Universidad Surcolombiana, Neiva, Huila, Colombia; ^3^ Dirección Laboratorio de Salud Pública, Secretaría de Salud Departamental, Gobernación del Huila, Neiva, Huila, Colombia; ^4^ Departamento de Medicina Interna, E.S.E. Hospital Universitario de Neiva. Programa de Medicina, Universidad Surcolombiana, Neiva, Huila, Colombia; ^5^ Servicio de Neumología, E.S.E. Hospital Universitario de Neiva. Programa de Medicina, Universidad Surcolombiana, Neiva, Huila, Colombia

**Keywords:** SARS-CoV-2, COVID-19, TGF-β1, IgM, IgA, IgG, RBD, spike

## Abstract

Identifying immune markers driving early and effective antibody response in patients with severe coronavirus disease 2019 (COVID-19) is critical due to the threat of future coronavirus pandemics, incomplete global vaccination, and suboptimal booster coverage. Patients with life-threatening severe acute respiratory syndrome coronavirus 2 (SARS-CoV-2) infection are characterized by dysregulated thromboinflammation and cytokine storm that could influence the isotype virus-specific antibody response and the subsequent clinical outcome. We investigated the association between COVID-19-related mortality with the dynamics, magnitude, and relative avidity of nucleoprotein (N), spike (S), and receptor-binding domain (RBD)-specific IgM, IgA, and IgG in circulation. We also assessed the relationship between the virus-specific antibody responses and cytokine patterns, as well as systemic and pulmonary thromboinflammation markers. This multicenter study included COVID-19 patients hospitalized early in the pandemic, classified as survivors (n=62) and non-survivors (n=17). We developed indirect enzyme-linked immunosorbent assays (ELISAs) to evaluate each virus-specific isotype using well-characterized outpatient COVID-19 (n=180) and pre-pandemic cohorts (n=111). The pro-inflammatory interleukin (IL)-6 and tumor necrosis factor (TNF)-α, as well as the regulatory IL-10, transforming growth factor (TGF)-β1, and soluble tumor necrosis factor receptor I (sTNFRI) levels were evaluated. The ELISAs performed highly for all virus-specific isotypes, although modest for IgM-N. Non-survivors increased N-specific, but no S-specific, IgM and IgA responses throughout the disease course and, more notably, a delayed class switching to IgG-S and IgG-RBD compared to survivors. No differences were observed in the virus-specific IgG relative avidity. Survivors exhibited an antibody response proportional to the degree of systemic and pulmonary thromboinflammation, whereas non-survivors showed those dissociated because of their uncontrolled severe thromboinflammation. Only the survivors showed a dominant regulatory cytokine pattern in the early phase of infection (<10 days after symptoms onset), which strongly correlated with developing IgG-S and IgG-RBD protective antibodies. We developed easy-to-use immune assays that enable patient monitoring and identify at-risk populations in low- to middle-income regions. Non-survivors displayed an ineffective N-mediated antibody response, marked by an inability to control inflammation and a compromised time-dependent class switching toward S and RBD-specific IgG. The regulatory cytokine axis, including TGF-β1, maybe a critical immune correlate of effective antibody-mediated immunity in COVID-19.

## Graphical Abstract

Unique virus-specific antibody characteristics distinguished survivors from non-survivors of COVID-19: non-survivors exhibited altered dynamic of S-specific IgM and IgA responses **(A)**, with a delayed instauration of S and RBD-specific IgG response **(B)**. The early development of these antibodies was associated with a robust regulatory cytokine response in survivors. Finally, non-survivors displayed a dissociated humoral response from systemic and pulmonary thromboinflammation **(C)**. Created in BioRender. Castro, S. (2025) https://BioRender.com/o07y756.

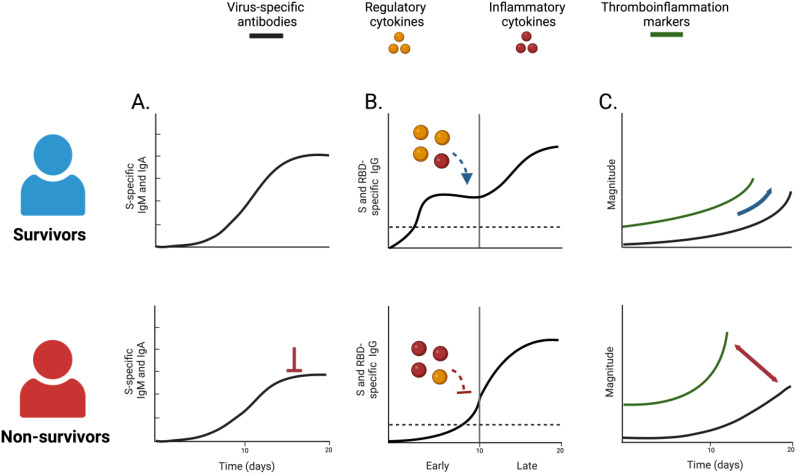

## 1 Introduction

Although the severe acute respiratory syndrome coronavirus 2 (SARS-CoV-2) vaccination has reduced the burden of coronavirus disease of 2019 (COVID-19), at least one-third of the world population remains unvaccinated, and many lack optimal booster coverage (1, 2). Identifying protective immune correlates to severity and mortality is essential for guiding clinical management, vaccine development, and pandemic preparedness as the threat of future coronavirus pandemics persists. The antibody response induced by SARS-CoV-2 infection is partially effective, lacking long-lasting protection similar to other respiratory viruses such as SARS-CoV, MERS-CoV, or Influenza (3). Given the rapid progression to a life-threatening condition—characterized by acute respiratory distress syndrome (ARDS) and systemic thromboinflammation—early immune responses may predict COVID-19 outcomes (4). Until now, the relationship between early antibody and cytokine responses driving disease outcomes has not been thoroughly characterized.

Following SARS-CoV-2 infection, most patients exhibited seroconversion within one to two weeks after symptom onset, marked by a simultaneous rise in virus-specific IgM, IgA, and IgG responses, with initial IgA predominance (5). The humoral response targets immunodominant structural viral proteins such as nucleoprotein (N) and spike (S). The S protein contains the receptor-binding domain (RBD) that mediates viral entry through the angiotensin-converting enzyme 2 (ACE2) receptor on host cells. Consequently, most of the neutralizing capacity relies on S and RBD-specific antibodies (6).

The effect of the antibody response on disease progression and mortality in COVID-19 remains controversial due to pathogenic and protective roles. In critically ill patients, aberrant afucosylation of SARS-CoV-2-specific antibodies increased immune-complex formation, leading to antibody-mediated pulmonary vascular inflammation and the release of inflammatory cytokines through Fc-receptors (7). Nucleoprotein-specific antibodies are associated with antibody-dependent enhancement and cross-reactivity to self-proteins, which may influence disease progression (8). Conversely, other studies showed that higher S-specific IgM and IgG antibody levels reduced mortality risk (9). The early development of these antibodies enhanced viral clearance and clinical outcomes (10). Other studies have found no such associations (11). One possible reason for these contradictory findings is the type and time of antibody response evaluated, as few have examined the relationship between multiple virus-specific antibody types and COVID-19-related mortality.

The cytokine responses are closely linked to the pathogenesis of COVID-19. An aberrant innate response (cytokine storm syndrome) is marked by dysregulated inflammatory cytokines such as interleukin (IL)-6, tumor necrosis factor (TNF)-α, and interferon (IFN)-γ. Indeed, circulating IL-6 levels can identify those who survived from those who did not survive COVID-19 (4). This response is accompanied by regulatory cytokines such as IL-10, transforming growth factor (TGF)-β1, and soluble tumor necrosis factor receptor I (sTNFRI) (12). The ability of the immunoregulatory cytokines axis to quickly counteract systemic inflammation distinguishes survivors from non-survivors of COVID-19 (4).

The interplay between inflammatory and regulatory cytokines could influence the antibody response. Specific cytokines such as IL-4, IL-6, TNF-α, IL-10, and TGF-β1 modulate the development, proliferation, and differentiation of B cells into antibody-secreting cells (13). Excessive TNF-α levels can hinder germinal center formation in severe and fatal SARS-CoV-2 infection (14, 15). The inflammatory cytokine response has been associated with decreased virus-specific IgG antibodies and mortality in COVID-19 (16). In contrast, the regulatory cytokine axis, including the TGF-β1, is vital for immune homeostasis, regulating inflammation, and modulating germinal center responses (17). The TGF-β1 signaling influences the humoral response and isotype-switching during severe COVID-19 (18). Thus, a balanced cytokine environment may fate antibody responses.

Previous research has examined virus-specific antibody responses associated with survival and the direct role of cytokines in pathogenesis. However, the association between the pattern of cytokines—including pro-inflammatory and regulatory types—and the early repertory of circulating antibodies and their impact on COVID-19-related mortality remains unexplored. In this multicenter study, we developed and validated indirect enzyme-linked immunosorbent assays (ELISAs) for detecting SARS-CoV-2-specific IgM, IgA, and IgG based on N, S, and RBD viral proteins. We investigated the association between the virus-specific antibody response characteristics (dynamic, magnitude, and avidity) and mortality in adults with COVID-19, as well as determined whether the pro-inflammatory or regulatory cytokine response modulates these antibody responses.

## 2 Patients and methods

### 2.1 Participants

Experiments followed the principles of the Declaration of Helsinki, and all study participants provided informed consent. This multicenter study was approved by the Ethics Committee of Hospital San Vicente de Paul, Garzón, Huila (Approval letter No. 014, 2020) and Hospital Universitario de Neiva, Huila (Approval letter No. 005-002, 2020). We included individuals older than 18 with respiratory symptoms (usually fever and dry cough) requiring hospitalization as COVID-19-suspected patients (n=100) between August and November 2020 in Neiva and Garzón, two cities in southern Colombia. Within 24 hours of hospital admission, a sample of 5 mL of peripheral venous blood was collected. The SARS-CoV-2 infection was confirmed using qualitative results (reported as positive or negative) of the real-time reverse transcriptase polymerase chain reaction (RT-qPCR) or flow lateral antigen test in nasopharyngeal swabs in concordance with diagnostic protocols of the local healthcare centers. Hospitalized confirmed COVID-19 patients (n=79) were classified as survivors (n=62) and non-survivors (n=17). During hospitalization, all included patients received steroids (dexamethasone 6mg/day) in concordance with the inpatient treatment protocols for moderate/severe COVID-19. The cohort’s clinical features, thromboinflammation markers, and cytokines plasma levels were previously characterized in an independent publication (4).

Hospitalized patients with COVID-19 were classified according to the days of symptoms into the early (< 10 days) or late (≥10 days) phase of infection. This allowed the evaluation of the virus-specific antibody dynamics. A second sample (convalescent) with a median (range) of 42 days (16–71) after symptom onset was taken from a subset of patients (n=18, including two non-survivors). Notably, in Colombia, vaccine distribution began in February 2021. Therefore, we evaluated the humoral response to SARS-CoV-2 after natural infection.

**Figure 1:**
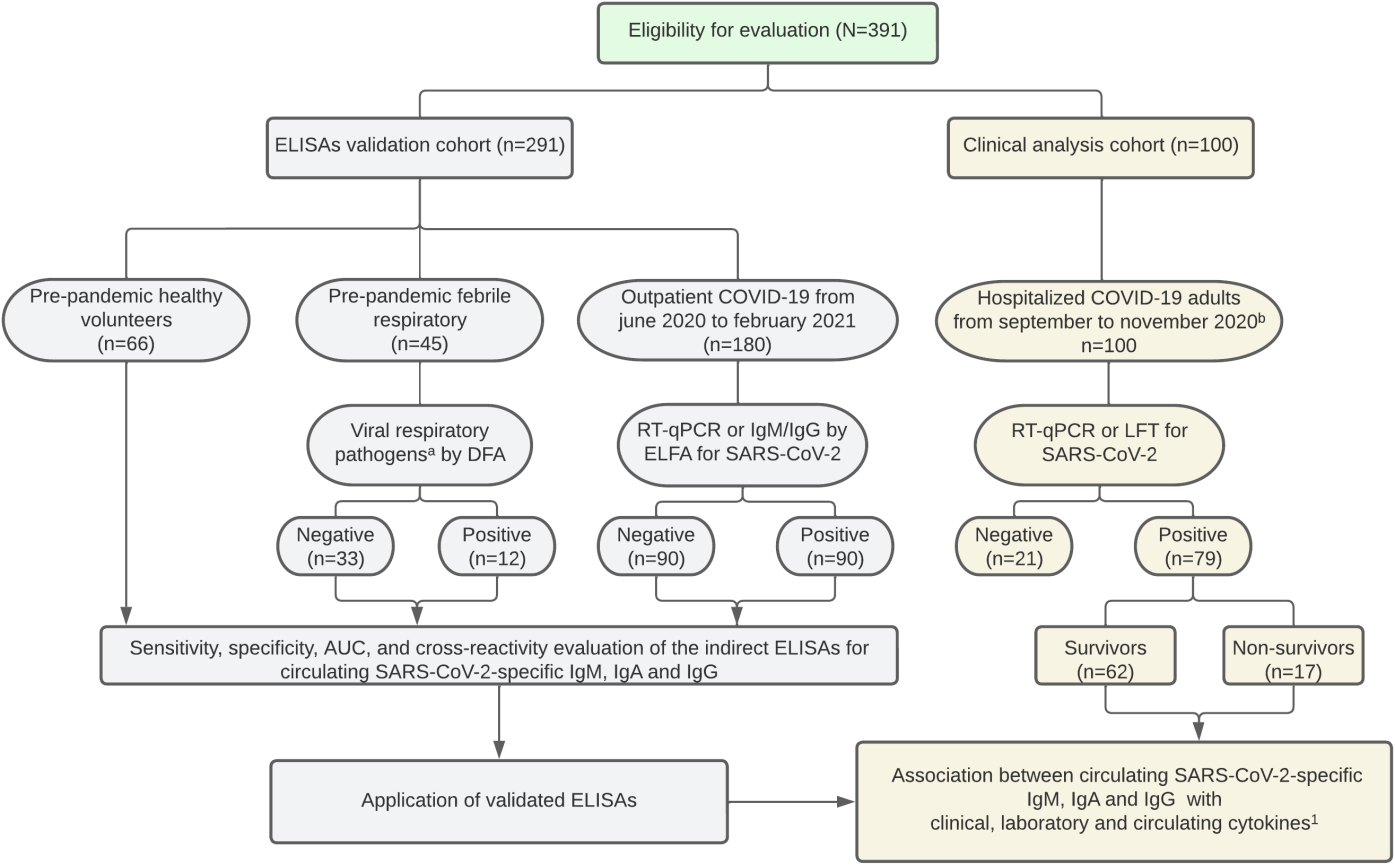
Study flow chart. ^a^ Influenza, Parainfluenza 1-2-3, and respiratory siyncitial virus were evaluated. ^b^ SARS-CoV-2 vaccine distribution began in February 2021 in Colombia. ELISA, enzyme-linked immunosorbent assay. DFA, direct fluorescence assay. ELFA, enzyme-linked fluorescence assay. RT-qPCR, reverse transcription-quantitative polymerase chain reaction. LFT, lateral flow test. ^1^Bolívar-Marín S, et al. A Specific Pattern and Dynamics of Circulating Cytokines Are Associated with the Extension of Lung Injury and Mortality in Colombian Adults with Coronavirus Disease-19. J Interf Cytokine Res. 2023; 43(5):206–215.

We included 291 patients to validate the indirect ELISAs for IgM, IgA, and IgG for N, S, and RBD viral proteins. A well-characterized outpatient COVID-19 cohort (n= 180) was enrolled between June 2020 and February 2021 in collaboration with Diagnosticamos, a private clinical laboratory in Neiva, Huila. The presence or absence of SARS-CoV-2-specific IgM and IgG was confirmed using enzyme-linked fluorescence assay (ELFA) with the commercial kits VIDAS® SARS-CoV-2 IgM (Biomérieux, catalog #: 423833-01, Marcy l’Étoile, France) and VIDAS® SARS-CoV-2 IgG (Biomérieux, catalog #: 423834-01, Marcy l’Étoile, France). Additionally, the SARS-CoV-2 envelope (E) gene was detected for diagnosis using RT-qPCR in a significant number of patients (n=66). To validate the virus-specific IgA ELISAs, we used samples with confirmed or discarded SARS-CoV-2 infection through RT-qPCR and ELFA (IgM and IgG). Moreover, we included negative control plasma from pre-pandemic healthy pediatric volunteers (n=66, collected between 2011-2014) and children with febrile respiratory illness (n=45, collected between 2012-2013). In the group with febrile respiratory illness, we looked at nasopharyngeal swabs of common respiratory viruses to evaluate the level of cross-reactivity in the ELISAs, using commercially available immunofluorescence-based kits for Influenza A and B (Reference: IFMINFA and IFMINFB, Vircell S.L., Granada, Spain), Parainfluenza 1, 2, and 3 (Reference: IFMPIV, Vircell S.L., Granada, Spain), and respiratory siyncitial virus (RSV) (Reference: IFMRSV, Vircell S.L., Granada, Spain). The study design is shown in **Figure 1**.

### 2.2 Indirect ELISA for SARS-CoV-2-specific IgM, IgA, and IgG

We evaluated the plasma virus-specific IgM, IgA, and IgG through a modified indirect ELISA based on SARS-CoV-2 recombinant proteins from the Wuhan-Hu-1 strain and purified from culture supernatant with a His-tag. The N (Expressed in Escherichia coli, Code: REC31851-500, Lot: 21041215, Accession: MN908947), S full-length (Expressed in Chinese hamster ovary [CHO] cells, Code: REC31868-500, Lot: 20112609D, Accession: YP_009724390.1), and RBD fragment (Expressed in HEK293 cells, Code: REC31882-500, Lot: 20112609D, Accession: 6XDG_E) were obtained from The Native Antigen Company, Kidlington, UK. Immulon 4 HBX 96-well plates (ThermoScientific, cat: 3855) were coated overnight at 4°C with 50 μL of N, S, or RBD at 1 μg/mL. Subsequently, the wells were blocked with 150 μL of 5% Blotto (skim milk powder [Chem Cruz, cat: SC-2325] in 1X phosphate-buffered saline [PBS] plus 0.1% Tween 20). After discharging, wells were incubated for 2 h at 37°C with 50 μL of diluted plasma 1:100 for IgM or 1:200 for IgA and IgG in 2.5% Blotto. After three washes with PBS 1X, 0.1% Tween 20 (wash buffer) was added 50 μL of biotin-labeled goat anti-human IgM (Seracare-KPL, cat: 161003), IgA (Seracare-KPL, cat: 161001), or IgG (Seracare-KPL, cat: 5260-0031) at 0.5 μg/mL in blotto 2.5%, followed by incubation for 1 h at 37°C. Then, 50 μL of 0.5 μg/mL streptavidin-peroxidase (Seracare-KPL, cat: 5270-0029) in blotto 2.5% was added and incubated for 1 h at 37°C. Finally, 50 μL of tetramethyl benzidine (Seracare-KPL, TMB cat: 51200049 and TMB component B cat: 51200038) was added. The reaction was stopped with 50 μL of 2M H_2_SO_4_ (Merck, Darmstadt, Germany; cat: 112080), and the wells were read at 450 nm in a Varioskan Lux multimode microplate reader (ThermoScientific, cat: N16706). All experiments included plasma from patients with confirmed SARS-CoV-2 infection through RT-qPCR and ELFA (IgM or IgG) as a positive control, as well as pre-pandemic healthy volunteers as a negative control. IgM anti-RBD was not evaluated.

### 2.3 Relative avidity assay

We evaluated the avidity of plasma N, S, and RBD-specific IgG antibodies through a relative avidity assay based on urea, as previously reported for dengue virus-specific IgG (19). Briefly, after the 2 h incubation of diluted plasma to wells coated with virus recombinant proteins, 50 μl of urea 8M (Catalog: M123-500G, Lot: 1883C451) or distilled water (control) was added, followed by incubation for 10 minutes at room temperature, and the subsequent steps reported for the SARS-CoV-2 IgG ELISA were then performed. The relative avidity index (expressed as a percentage) was evaluated by the ratio of the absolute optical density (OD) at 450 nm of the treated sample (urea 8M) over the non-treated sample (distilled water).

### 2.4 Association between the virus-specific antibody response with the thromboinflammation markers and cytokines

Through correlation analysis, we assessed the relationship of the virus-specific antibody responses with the systemic and pulmonary severity markers, as well as circulating cytokines previously well-characterized in the COVID-19 hospitalized cohort (4). We included the severity markers D-dimer, ferritin, lactate dehydrogenase (LDH), C-reactive protein (CRP), neutrophil-to-leucocyte ratio (NLR), arterial-to-inspired oxygen (PaO_2_/FiO_2_), and the extent of lung injury using an axial computed tomography (CT)-based severity score (20). We also evaluated whether virus-specific responses are modulated by circulating pro-inflammatory (IL-6 and TNF-α) and regulatory (IL-10, TGF-β1, and sTNFRI) cytokine responses. Although plasma levels of IFN-γ, IL-4, and the Thymic stromal lymphopoietin (TSLP) were also evaluated, these results are not presented as most were under detection limit or showed no significant association with the virus-specific humoral response.

### 2.5 Statistical analysis

The results are presented as median and range. Statistical analysis was performed with GraphPad Prism 9 for MAC, GraphPad Software, Boston, Massachusetts, USA. The Mann-Whitney and Kruskal-Wallis tests were used to determine differences between two or more independent groups. If the p-value of the Kruskal–Wallis test was <0.05, Dunn’s *post hoc* test was performed. Paired analyses of two groups were performed with the Wilcoxon test. Differences in the expected frequency of an event were evaluated using the Fisher exact test. The Spearman rank test and linear regression analysis determined the correlation between variables. To assess the performance of the indirect ELISA for SARS-CoV-2 N, S, and RBD-specific immunoglobulins, the receiver operating characteristic (ROC) curve analysis was used. The grade of concordance among different assays is presented as the agreement and the Cohen Kappa Index. The cutoff points were established based on the highest Youden J Index for the validation analysis (21), and the cutoff points shown in the clinical analysis for each immunoglobulin isotype were established based on the average of N, S, and RBD-specific response. In all cases, p < 0.05 was used to determine significance.

## 3 Results

### 3.1 Epidemiological characteristics of the study populations

Two hundred ninety-one individuals were analyzed to evaluate the performance of the SARS-CoV-2-specific immunoglobulins ELISAs (**Table 1**). Pre-pandemic healthy and febrile respiratory subjects were younger than pandemic patients with or without SARS-CoV-2 infection, as these were included in the pediatric department during local respiratory epidemics.

**Table 1:** Epidemiological characteristics of the study population.

	Validation					Hospitalized COVID-19		
	Pre-pandemic healthy	Pre-pandemic febrile respiratory	Outpatient COVID-19			Survivors	Non-survivors	
Parameter	n=66	n=45	Negative n=90	Positive n=90	p-value	n=62	n=17	p-value
Age (years), median (range)	13 (11-16)	3 (0.2-5)	42 (7-82)	47 (8-78)	<0.001^a^	44 (15-84)	68 (36-91)	< 0.001^b^
Female/male (%)	47/53	48/52	55/45	41/59	0.359^c^	55/45	18/82	0.012^c^
Days after symptoms onset, median (range)	**-**	3 (2-16)	19 (13-104)*	18 (1-101)*	<0.001^a^	7 (1-23)	11 (1-22)	0.087^b^
NLR	**-**	**-**	**-**	**-**		7 (0.75-32)	10 (2.8-70)	0.060^b^
CT-severity score	**-**	**-**	**-**	**-**		13 (0-20)	18 (4-20)	<0.001^b^

a Kruskal–Wallis test.

b Mann-Whitney test.

c Fisher´s exact test, COVID-19, coronavirus disease-19; NLR, neutrophil-to-leucocyte ratio; CT, computed tomography; *Days after diagnosis is shown. p <0.05 was considered significant.

The hospitalized COVID-19 group was classified as survivors (n=62 [78.5%]) and non-survivors (n=17 [21.5%]). The non-survivor group was older and exhibited a higher male proportion than the survivor group (**Table 1**), consistent with previous reports. Life-threatening COVID-19 is characterized by systemic thromboinflammation and ARDS-associated lung damage. As expected, non-survivors had significantly higher levels of D-dimer, ferritin, LDH, and CRP than survivors. A trend to higher NLR values in non-survivors was observed (**Table 1**). Additionally, non-survivors displayed significantly lower levels of PaO_2_/FiO_2_ and higher CT scores, indicating more severe lung damage. These patients’ complete clinical characteristics and laboratory tests are detailed elsewhere (4).

### 3.2 Performance of the indirect ELISAs for SARS-CoV-2-specific IgM, IgA, and IgG

**Figure 2:**
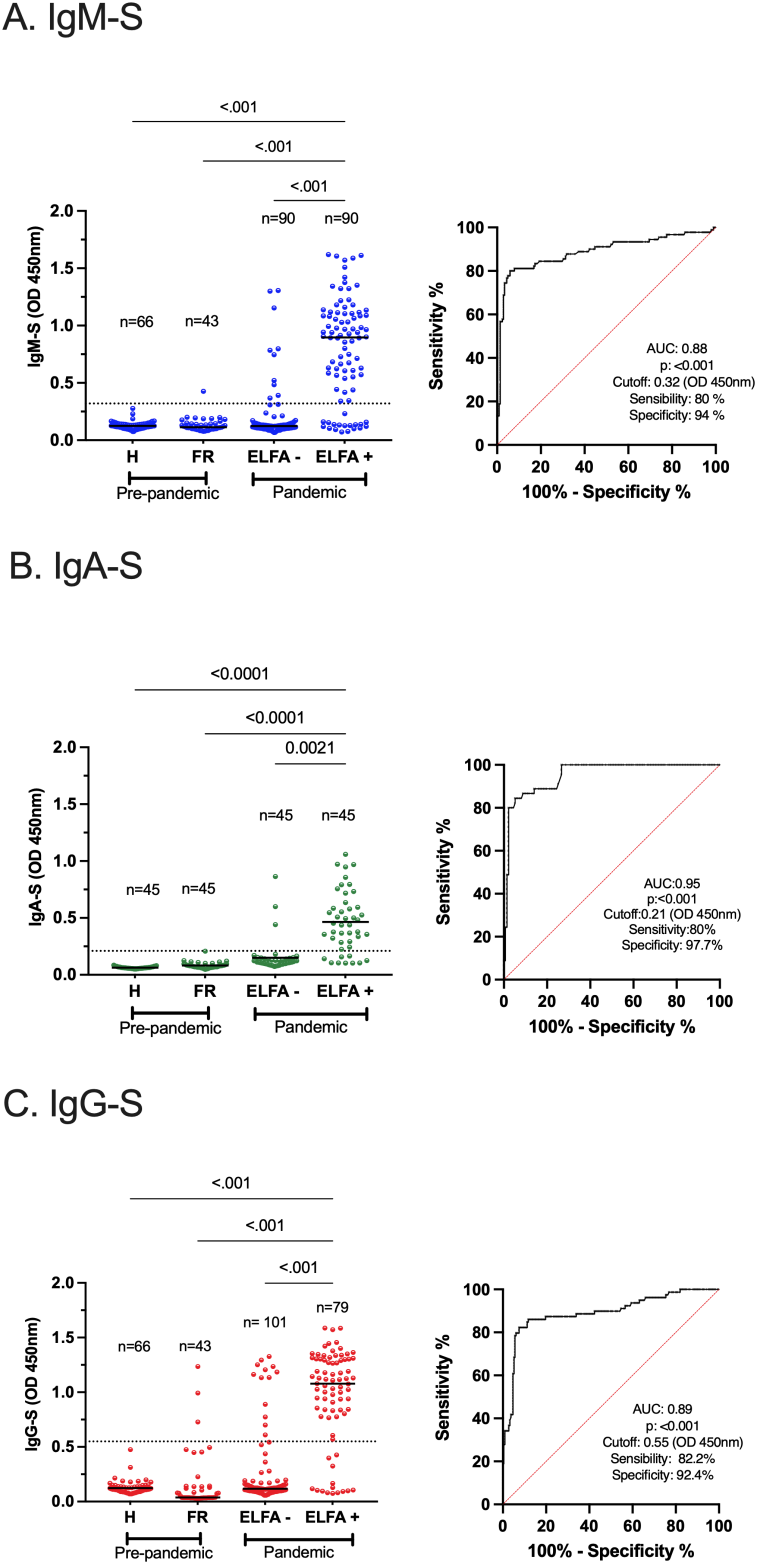
Performance of indirect ELISAs for SARS-CoV-2-specific IgM, IgA, and IgG. Analysis of plasma samples from outpatient individuals with SARS-CoV-2 infection confirmed by RT-qPCR and SARS-CoV-2-specific IgM and IgG through a commercially available ELFA approved for human diagnosis **(A–C)** left. As a negative control, pre-pandemic samples from healthy (H) and febrile respiratory (FR) subjects were included. In the FR group, the overall cross-reactivity was evaluated. The extended analysis of all virus-specific isotype responses is shown in **Supplementary Figure 1**. The ROC curve analysis of each S-specific antibody response is shown, with the area under the curve (AUC), p-value, cutoff, sensitivity, and specificity **(A–C)** right. The dotted lines illustrate the cutoff values based on the highest Youden J Index. The Kruskal–Wallis test was applied for the analysis between groups, and Dunn’s *post hoc* test was performed. p <0.05 was considered significant. RT-qPCR, reverse transcriptase–quantitative polymerase chain reaction, ELISA, enzyme-linked immunosorbent assay, ELFA, enzyme-linked fluorescence assay, ROC, receiver operating characteristic.

A set of indirect ELISAs was validated to detect virus-specific IgM, IgA, and IgG based on N, S, and RBD proteins. We included samples of outpatient adults with confirmed or ruled-out SARS-CoV-2 infection through RT-qPCR and commercial ELFA detecting virus-specific IgM and IgG. As negative controls, pre-pandemic samples (collected between 2011 and 2014) were also included from healthy and children with symptomatic respiratory infections. The ELISAs performance for S-specific IgM, IgA, and IgG is shown in **Figure 2** and extended for each viral protein and isotype-specific antibody in **Supplementary Figure 1**. The ELISAs performance is summarized in **Table 2**. Overall, immune assays for virus-specific IgM, IgA, and IgG showed a range of sensitivity and specificity from 80 to 88% and 70 to 97%, respectively. A particular case was the IgM-N assay low-sensitivity (**Table 2**). The plasma levels of N, S, and RBD-specific IgG increased from acute to convalescence paired clinical samples, corroborating the performance of the validated assays for detecting seroconversion (**Supplementary Figure 2**).

**Table 2:** Performance of SARS-CoV-2-specific IgM, IgA, and IgG assay.

Virus-specific antibodies	Cross-reactivity (%)	Agreement (%)	Kappa index	Sensitivity (%)	Specificity (%)	PPV (%)	NPV (%)	AUC	p-value
IgM-N	9.3	76	0.46	67.7	79.9	67	79	0.76	<0.001
IgM-S	2.3	89.9	0.76	80	94	80	94	0.88	<0.001
IgA-N	4.4	74	0.45	86.6	70.3	86	70.4	0.87	<0.001
IgA-S	0	93.3	0.81	80	97.7	80	97	0.95	<0.001
IgA-RBD	2.2	85	0.63	84.4	85.1	84	85	0.93	<0.001
IgG-N	4.4	90	0.76	88.9	90.3	88.8	91.1	0.93	<0.001
IgG-S	16.2	89.6	0.74	82.2	92.4	82	92.3	0.89	<0.001
IgG-RBD	13.9	88.9	0.73	84.8	90.5	84	90.4	0.88	<0.001

PPV, positive predictive value; NPV, negative predictive value; AUC, area under the curve; ROC, receiver operating characteristic curve analysis. p <0.05 were considered significant.

We applied the virus-specific IgM, IgA, and IgG assays in the hospitalized COVID-19 group. As expected, the virus-specific antibody responses increased significantly over time (**Supplementary Figures 3A–C**), with an early predominance of the virus-specific IgA over IgG responses, followed by a late change to IgG predominance (**Supplementary Figure 3D**). These results agreed with previous reports showing the fast induction of plasma SARS-CoV-2 specific IgA response after natural infection (5).

Pre-existing humoral immunity to other human coronaviruses or common respiratory viruses causing the common cold has been implicated in disease progression in COVID-19 (22). To mitigate potential bias, we assessed the overall levels of cross-reactivity of our indirect ELISAs in a febrile respiratory pre-pandemic pediatric group. Although we did not evaluate other coronaviruses, we confirmed 26% (12/45) cases of respiratory virus infection through DFA: influenza virus (2/12), parainfluenza virus (2/12), and RSV (8/12). Hence, a mild frequency of overall cross-reactivity was observed in the IgM-N and IgA-N responses (**Table 2**), possibly due to the conserved regions of N protein among respiratory viruses or previously circulating coronavirus (23). Although we observed IgG-S and IgG-RBD cross-reactivity compared to IgG-N (**Table 2**), this was not evidenced in the pre-pandemic healthy samples (**Supplementary Figures 1D** left, **E** left). Thus, the indirect ELISAs displayed low levels of cross-reactivity, particularly for anti-N and anti-S responses.

### 3.3 Altered dynamics of S-specific IgM and IgA responses are associated with COVID-19-related mortality

**Figure 3:**
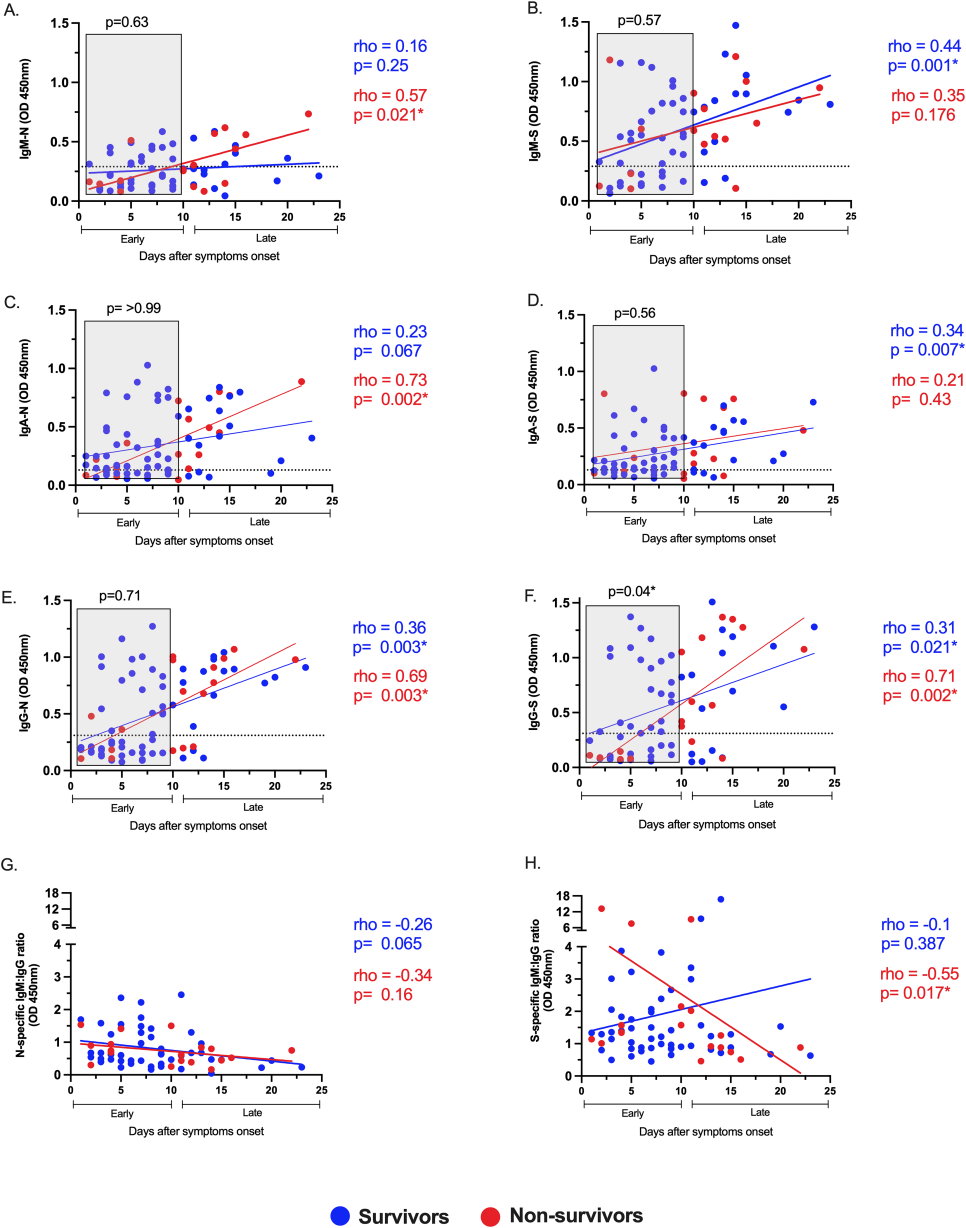
Altered dynamics of S-specific IgM and IgA and delayed IgG-S instauration are associated with COVID-19-related mortality. Correlation and linear regression analyses of the virus-specific responses IgM, IgA, and IgG for N **(A, C, E)** and S **(B, D, F)** viral proteins, respectively, and the ratio IgM to IgG for N **(G)** and S **(H)** proteins, with days after symptom onset in survivors (blue circles) and non-survivors (red circles) of COVID-19. The Spearman test *rho* and p-values are color-coded (blue for survivors, red for non-survivors). The dotted lines indicate the cutoff value. COVID-19 patients were classified into early (<10 days) or late (≥10 days) phases post-symptom onset. The relative levels of the virus-specific antibody responses during the early phase, marked in gray boxes **(A–F)**, were compared between survivors and non-survivors. p-values from the Mann–Whitney test are shown above each gray box. p <0.05* was considered significant across all analyses.

Then, we investigated differences between survivors and non-survivors, evaluating the magnitude, dynamic, and relative avidity of the SARS-CoV-2 isotype-specific antibody response. The dynamic was assessed by correlating virus-specific IgM, IgA, and IgG plasma levels with the days after symptom onset (**Figure 3**). The antibody response displayed unique dynamics depending on the disease outcome. Survivor patients significantly increased their S-specific but not N-specific IgM and IgA levels over time. Conversely, non-survivors exhibited an opposite pattern for S-specific IgM and IgA (both showed no increase), as well as a significant increase for N-specific IgM and IgA responses (**Figures 3A–D**). The N and S-specific IgG levels increased significantly over time regardless of clinical outcome (**Figures 3E, F**). These results indicate that the evolution of the S-specific instead of N-specific IgM and IgA responses may impact the survival of COVID-19.

### 3.4 Delayed S and RBD-specific IgG response is linked to COVID-19-related mortality

The presence of early distinctive virus-specific antibodies may modulate the clinical outcome during SARS-CoV-2 infection (24). Thus, we divided the antibody response into early (< 10 days) or late (>10 days) phases according to the days after symptoms onset. Globally, the early plasma levels of virus-specific IgM, IgA, and IgG were compared between survivors and non-survivors (**Figure 3** Gray box in A–F). Notably, the early IgG-S response was higher in those who survived than those who did not, and this difference was not evident for all other virus-specific antibodies evaluated, suggesting that early development of IgG-S antibodies is critical for recovery from COVID-19. Consistent with this, we used the IgM/IgG isotype ratio for N and S to evaluate the predominant isotype for each viral protein over time (**Figures 3G, H**). In the S-specific response, non-survivors displayed an early IgM predominance to IgG, no further observed at the late stage (rho=-0.55, p= 0.017, **Figure 3H**), revealing a delayed switch class to IgG compared to survivors. In contrast, there was no isotype predominance in the N-specific response through time, neither in survivors nor non-survivors (**Figure 3G**). These results suggest that a dysfunctional IgG-S response in a time-dependent manner is associated with COVID-19-related mortality.

We also observed an early IgG-RBD higher response in the survivors compared to the non-survivors group (**Supplementary Figure 4**. Gray box in A). Of note, the S-specific antibodies elicited during SAR-CoV-2 infection can be reactive to RBD due to their structural relationship but have differential functions (25). Consequently, the IgG-S plasma levels strongly correlated with the IgG-RBD levels but to a lesser extent with IgG-N (**Supplementary Figures 4B, C**). Furthermore, we assessed the functionality of these antibodies through relative avidity changes over time in the COVID-19 hospitalized group with a convalescent sample (**Supplementary Figure 5A**). We observed affinity maturation only in the IgG-RBD response, indicating that our indirect ELISA can identify specific changes in affinity maturation, which RBD primarily drives (26). The IgG-RBD relative avidity was higher in those with more severe disease in the convalescent (**Supplementary Figure 5B**). Conversely, we found no differences in the relative avidity of IgG-N, S, and RBD between survivors and non-survivors in the acute phase of infection (**Supplementary Figure 5C**), in concordance with the low somatic hypermutations observed in acute sera from COVID-19 patients (27). Together, these findings suggest that the maturation of the virus-specific antibody response takes place in later stages following acute infection, but the early development of IgG-S and IgG-RBD antibodies might prevent COVID-19-related mortality.

### 3.5 Dissociated antibody response from systemic and pulmonary thromboinflammation is associated with COVID-19-related mortality

**Figure 4:**
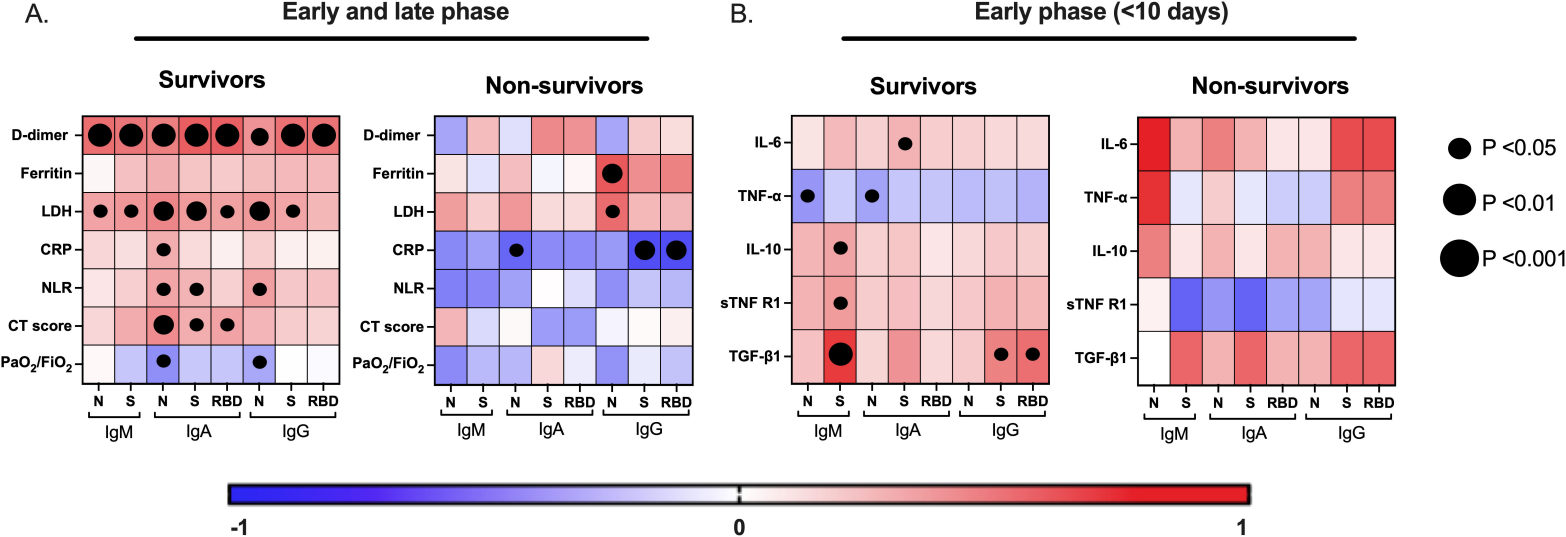
A distinctive correlation of the virus-specific antibody responses with severity markers and cytokines is associated with COVID-19-related mortality. The correlation matrix shows the virus-specific antibody responses (IgM, IgA, and IgG) with systemic/pulmonary severity markers of infection **(A)** and with proinflammatory and regulatory cytokine responses in the early (<10 days after symptoms onset) phase of the disease **(B)**, in survivors and non-survivors of COVID-19. LDH, lactate dehydrogenase, CRP, C-reactive protein, NLR, neutrophil-to-leucocyte ratio, PaO_2_/FiO_2_, arterial-to-inspired oxygen, computed tomography (CT)-based severity score.

The levels of D-dimer, LDH, CRP, NLR, and ferritin reflect the degree of systemic tromboinflammation and disease severity in COVID-19 patients. Similarly, the CT scan score and PaO_2_/FiO_2_ ratio indicate the extent of injury and dysfunction in the lung tissue, as well as peripheral hypoxia (4). Based on the clinical outcome, we evaluated the correlation between these severity markers and the virus-specific antibody levels in plasma (**Figure 4A**). Interestingly, D-dimer levels strongly correlated with all virus-specific antibody responses evaluated in the survivor group (**Figure 4A**, left). In contrast, no such correlation was observed in the non-survivor group (**Figure 4A**, right). A similar pattern was noted for LDH, CRP, and NLR. Therefore, the non-survivor virus-specific antibody response dissociated from the systemic thromboinflammation. This is further supported by the strong negative correlation between the CRP levels and the virus-specific antibody response in non-survivors (**Figure 4A**, right). The dissociation pattern of the antibody response was also observed with the pulmonary severity markers. The CT scan score and the PaO_2_/FiO_2_ ratio correlated with the virus-specific IgA response in the survivor group but not in the non-survivor group. Additionally, these results suggest that the circulating virus-specific IgA may come from a mucosal lung immune response. In conclusion, non-survivors exhibited a dissociated antibody response from systemic and pulmonary thromboinflammation, possibly due to their inability to control the severe inflammation.

### 3.6 Regulatory cytokines modulate the early development of virus-specific antibodies associated with survival in COVID-19

The ability of the immunoregulatory cytokines axis, particularly TGF-β1 and IL-10, to quickly counteract systemic inflammatory cytokines such as IL-6 is a hallmark in survivors relative to non-survivors of COVID-19 (4). Furthermore, given that cytokines are critical modulators of antibody-mediated immunity, we investigate whether the pro-inflammatory (IL-6 and TNF-α) and regulatory (IL-10, TGF-β1, and sTNFRI) cytokine responses influence the development of the virus-specific antibody response during the early stage of infection, in survivors and non-survivors of COVID-19 (**Figure 4B**). Notably, in survivors, the regulatory cytokine axis response positively correlated with the IgM-S levels, while the IgG-S and IgG-RBD—the key antibodies associated with COVID-19 survival—correlated strongly with the TGF-β1 response. This pattern was not observed in the other virus-specific antibody responses (**Figure 4B**, left). In contrast, no correlations were found in the non-survivor group, but we observed a positive correlation trend between IL-6 and IgM-N levels (p= 0.08) (**Figure 4B**, right). These findings suggest that the regulatory cytokine axis, particularly the TGF-β1, favored an effective S and RBD antibody-mediated immunity associated with survival in COVID-19.

## 4 Discussion

Given the threat of future coronavirus pandemics, incomplete global distribution, and suboptimal booster coverage for the COVID-19 vaccine, it is necessary to identify early protective immune correlates from life-threatening disease. Here, using our validated indirect ELISAs, we identified distinctive characteristics of the virus-specific antibody response associated with COVID-19-related mortality. First, non-survivors exhibited increased N-specific, but no S-specific, IgM and IgA responses over the disease course, with delayed IgG-S and RBD-specific responses. Second, survivors exhibited a virus-specific antibody response correlated with thromboinflammation, while non-survivors presented these dissociated. Third, a regulatory cytokine response, particularly the TGF-β1, modulates the early development of IgM-S, IgG-S, and IgG-RBD antibodies associated with survival.

The antibody response balance against different SARS-CoV-2 structural proteins might impact the survival of COVID-19. The treatment with immunized sera against S and RBD proteins, but no N protein, protected against SARS-CoV-2 challenge in a mouse model (28). This is consistent with the strong correlation between S or RBD antibodies with virus neutralization and the inverse correlation of seroconversion with viremia (25). A shift in the humoral response toward N instead of S or RBD proteins could affect viral clearance. Correspondingly, Atyeo C et al. found that survivors exhibited higher antibody levels against S than N compared to non-survivors (29). Our findings support this, as non-survivors increased their N-specific, but no S-specific, IgM and IgA responses over the disease course. Thus, a divergence in virus-specific antibody repertory may affect the immune response effectiveness and disease outcomes.

In addition to the imbalance of humoral response to the N over S and RBD protein in non-survivors, the role of the N-specific antibody could be harmful: N-specific antibodies are reactive to self-proteins, affecting disease progression (8), and higher IgG-N levels predicted poor outcomes (30). However, it is noteworthy that the N protein is currently an attractive target for vaccine design due to its greater immunogenicity and homology among SARS-CoV-2 variants of concern (VOCs) compared to S and RBD proteins, which limits immune escape (23). Thus, the role of N-specific antibodies in disease outcomes remains unclear, and future studies must evaluate their usefulness in vaccine-induced immunity.

The role of the virus-specific antibody response in predicting disease outcomes may be time-dependent. Transfusion of convalescent plasma with high S-specific antibody levels reduced mortality risk. Notably, patients who received this immune plasma earlier had a lower risk of death compared to those who received it later in the disease course (31). While IgM and IgA isotypes have neutralizing effects—particularly dimeric IgA in local responses—IgG is the most potent circulating isotype for neutralizing SARS-CoV-2 (32). Multiple studies have reported that early neutralizing antibodies or S and RBD-specific IgG are protective correlates against COVID-19-related mortality (29, 33, 34). Therefore, non-survivors displayed a delayed rather than lower protective response (24). Non-neutralizing S-specific antibody properties have also been linked to viral spread control and survival, including increased phagocytosis and complement activation (29). However, other studies have found that S and RBD-specific IgA levels were higher in non-survivors, whereas N-specific IgA and IgG were associated with survival (35). Our findings support that a dysfunctional S and RBD-specific humoral response in a time-dependent manner may contribute to poor outcomes. Given that somatic hypermutation is not essential for the development of neutralizing antibodies against SARS-CoV-2 (36), the most likely protective mechanism behind these findings is the neutralizing capacity of these early IgG-S and RBD antibodies, as their levels are a strong correlate of neutralizing activity.

Germinal center reactions, which are essential for inducing appropriate antibody responses, isotype class switching, and affinity maturation, are modulated by antigen-specific T follicular-helper (Tfh) cells. These reactions seemed impaired in the early stages of severe SARS-CoV-2 infection (37), which likely explains the lack of association between virus-specific IgG avidity and disease outcomes in our cohort, supported by the low levels of somatic hypermutations observed in acute sera from COVID-19 patients (27). The proposed mechanism behind these impaired antibody responses is a defect in Tfh cell differentiation caused by excessive TNF-α levels, which hinder germinal center formation (14). Other inflammatory cytokines, such as IFN-γ and IL-6, have also been implicated, though the role of regulatory cytokines has not been thoroughly evaluated.

Our findings indicate that an appropriate systemic regulatory cytokine response, including TGF-β1, modulates the development of virus-specific antibodies associated with survival of COVID-19. The TGF-β signaling contributes to the development of influenza-specific Tfh cells, germinal center reactions, and isotype-switched flu-specific antibody responses (38). In line with this, the TGF-β signaling contributes to early IgG and IgA switch class in severe SARS-CoV-2 infection (18). Therefore, the interplay between inflammatory and regulatory cytokines may affect the antibody responses, where a predominance of inflammatory signaling in non-survivors could be related to dysfunctional induction and generally compromised antibody response unable to counteract or follow systemic/pulmonary inflammation. The negative effect on germinal center reactions may lead to unregulated extrafollicular B cell responses (39). Although these responses are an essential source of early isotype switch-class neutralizing antibodies in COVID-19 (40), what modulates these transitional responses remains largely unknown.

Cumulative evidence points to older age and male gender as risk factors for poor outcomes in COVID-19, which is in line with our findings. Still, host characteristics such as age may affect immune responses against SARS-CoV-2, possibly related to the immunosenescence (41). Furthermore, the male gender is associated with altered cellular and humoral responses in SARS-CoV-2 infection (42). These natural host properties may also affect other immune mechanisms, including T-cell-specific responses, which could be the surrogate of protective early antibodies against poor outcomes (43).

We developed and validated easy-to-use indirect ELISAs for detecting SARS-CoV-2-specific IgM, IgA, and IgG against the N, S, and RBD proteins. These assays demonstrate acceptable performance and support the diagnostic utility of various viral proteins, particularly for low-middle incoming regions (44, 45). Serological assays have broader applications beyond diagnosis, including epidemiological surveillance, evaluating vaccine efficacy, and identifying at-risk populations with inadequate or waning humoral immunity. Older patients and those with comorbidities or undergoing immunosuppressive treatment have been associated with insufficient humoral responses to infection or vaccination (46). High S-specific antibody levels at hospital admission are linked to survival in unvaccinated and vaccinated COVID-19 patients (47). Therefore, easy-to-use serological assays can help evaluate protective titer and appropriate booster regimens. In addition, the mucosal IgA response is an immune marker of protection against severe COVID-19 and strongly correlates with plasma IgA. However, most current vaccine designs overlook mucosal immune activation (39). Thus, IgA-based assays may serve as a correlate of vaccine-induced immunity in future vaccine designs involving mucosal immunity.

The limitations of our study include small sample size, absence of viral determinants such as plasma viral load, lack of functional assays for antibodies (neutralizing potential), longitudinal analysis of humoral responses between groups, and the lack of assessment of chemokines (e.g., CCL2, CXCL9, CXCL13) that modulate humoral responses. Consequently, further studies should investigate the protective role of early distinctive antibodies and conduct mechanistic studies to elucidate the complex interplay between inflammatory and regulatory cytokines—particularly the potential role of TGF-β signaling—in modulating antibody responses in severe SARS-CoV-2 infection. Additionally, while pre-existing humoral immunity to other coronaviruses lacks SARS-CoV-2 neutralizing capacity, its potential role in the early humoral responses should be considered (22).

In summary, we developed easy-to-use immunoassays that detect highly dynamic virus-specific humoral responses, enabling patient monitoring and identifying at-risk populations. Our findings suggest that the dysfunctional characteristics of this humoral response linked to mortality could be due to the interplay between inflammatory and regulatory cytokine responses, where a predominance of the regulatory axis, including TGF-β, is a possible crucial correlate of effective antibody-mediated immunity.

## Data Availability

The raw data supporting the conclusions of this article will be made available by the authors, without undue reservation.
