# Supplementary material for: Regulatory cytokines modulate early isotype-specific response associated with COVID-19 survival
Source: Front Immunol. 2025 Apr 24;16:1543626. doi: 10.3389/fimmu.2025.1543626 (PMC12058664; doi:10.3389/fimmu.2025.1543626)
Supplement: Supplementary file 1 [file DataSheet1.docx]

Supplementary Material

**Supplementary Figure 1.**

**
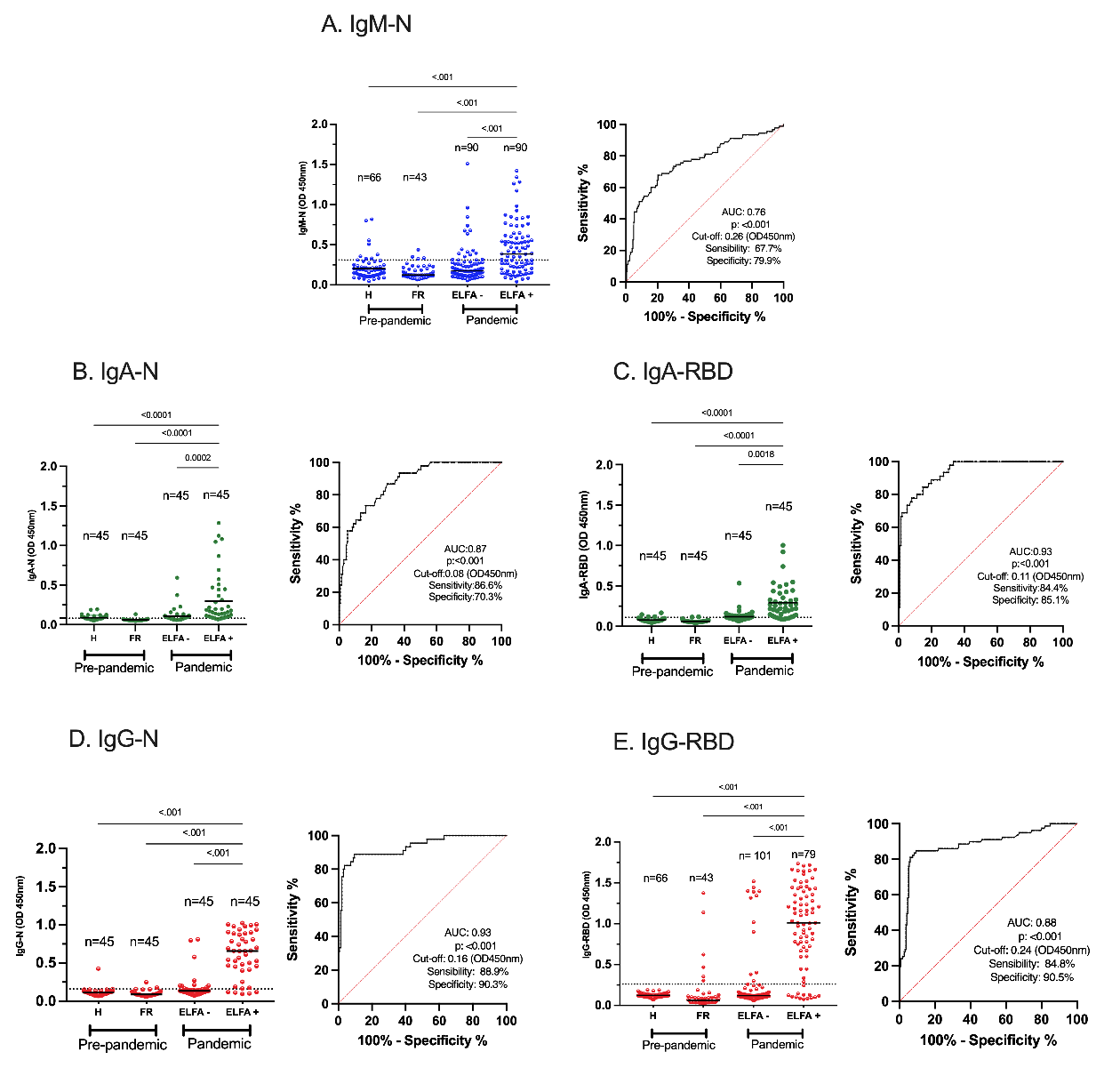
**

**Supplementary Figure 1. Performance of indirect ELISAs for SARS-CoV-2-specific IgM, IgA, and IgG.** Analysis of plasma samples from outpatient individuals with confirmed SARS-CoV-2 infection by RT-qPCR and virus-specific antibody response through ELFA **(A-E left)**. As a negative control, pre-pandemic samples from healthy (H) and febrile respiratory (FR) subjects were included. In the FR group, the overall cross-reactivity was evaluated. The ROC curve analysis of each virus-specific antibody response is shown, with the area under the curve (AUC), p-value, cutoff, sensitivity, and specificity **(A-E right)**. The dotted lines illustrate the cutoff values based on the highest Youden J Index. The Kruskal–Wallis test was applied for the analysis between groups, and Dunn’s post hoc test was performed. P <0.05 was considered significant. RT-qPCR, reverse transcriptase–quantitative polymerase chain reaction, ELISA, enzyme-linked immunosorbent assay, ELFA, enzyme-linked fluorescence assay, ROC, receiver operating characteristic.

**Supplementary Figure 2.**

**
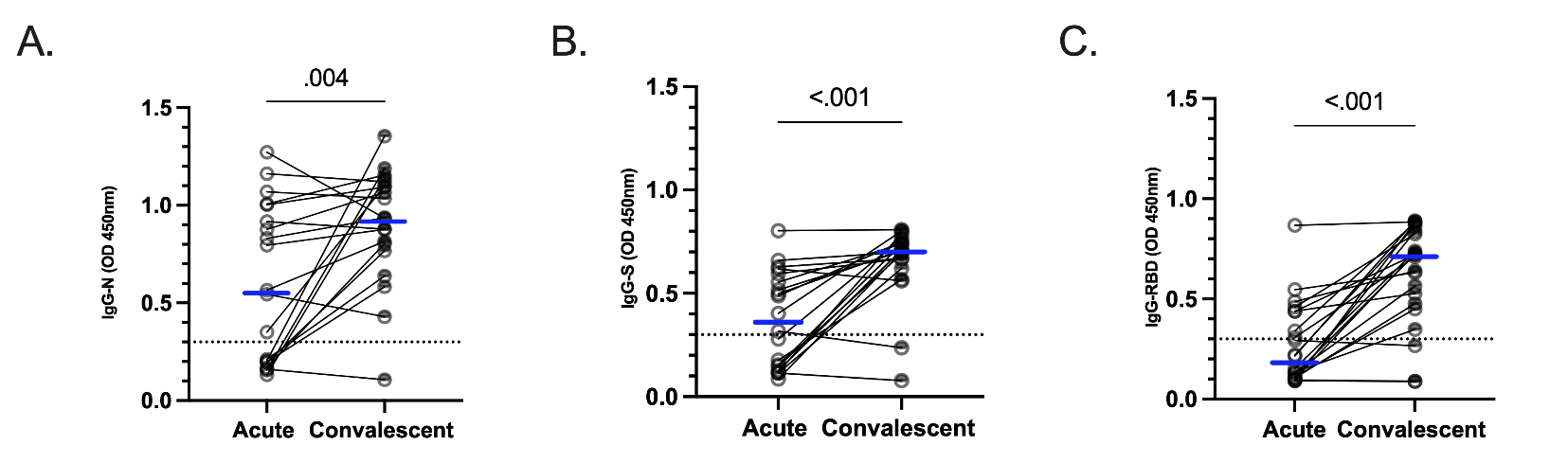
**

**Supplementary Figure 2. Seroconversion of SARS-CoV-2-specific IgG.** The relative plasma levels of N (A), S (B), and RBD (C) specific IgG response during the acute and convalescent phase of infection from hospitalized COVID-19 patients. p values from the Wilcoxon test are shown. The dotted lines indicate the cutoff value. p <0.05 was considered significant across all analyses.

**Supplementary Figure 3.**

**
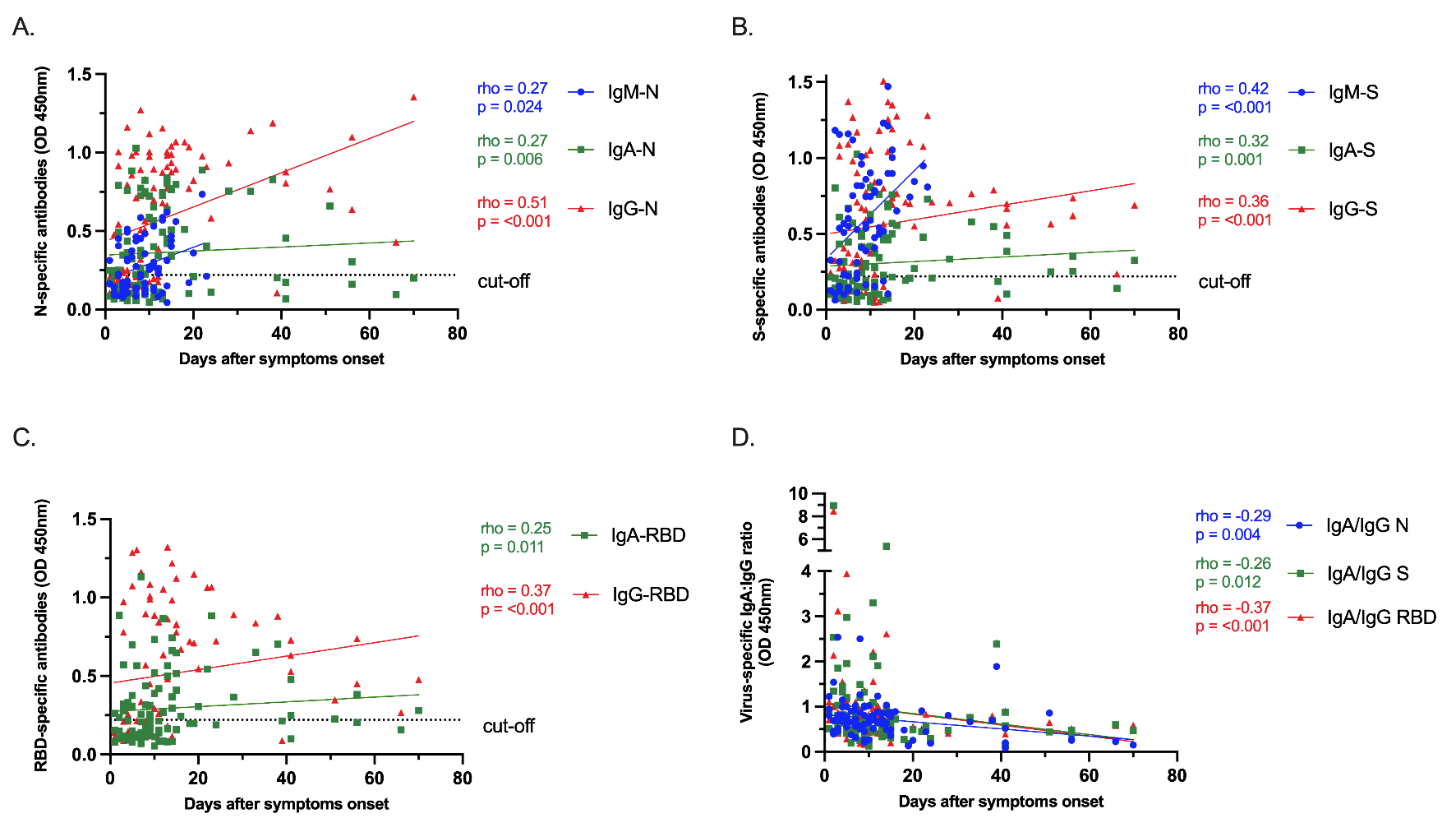
**

**Supplementary Figure 3. Dynamic of the SARS-CoV-2-specific IgM, IgA, and IgG responses.** Correlation and linear regression analyses of the virus-specific responses IgM, IgA, and IgG for N **(A)**, S **(B),** and RBD **(C)** proteins, respectively, in relation to the days after symptom onset in hospitalized COVID-19 patients. A second available sample (n=18) was included only for virus-specific IgA and IgG responses. The Spearman test rho and p values are color-coded: blue for IgM, green for IgA, and red for IgG. Correlation and linear regression analyses of IgA to IgG ratio for N, S, and RBD proteins **(D)** in relation to the days after symptom onset. The Spearman test rho and p values are color-coded: blue for IgA/IgG-N, green for IgA/IgG-S, and red for IgA/IgG-RBD. The dotted lines indicate the cutoff value. p <0.05 was considered significant across all analyses.

**Supplementary Figure 4.**

**
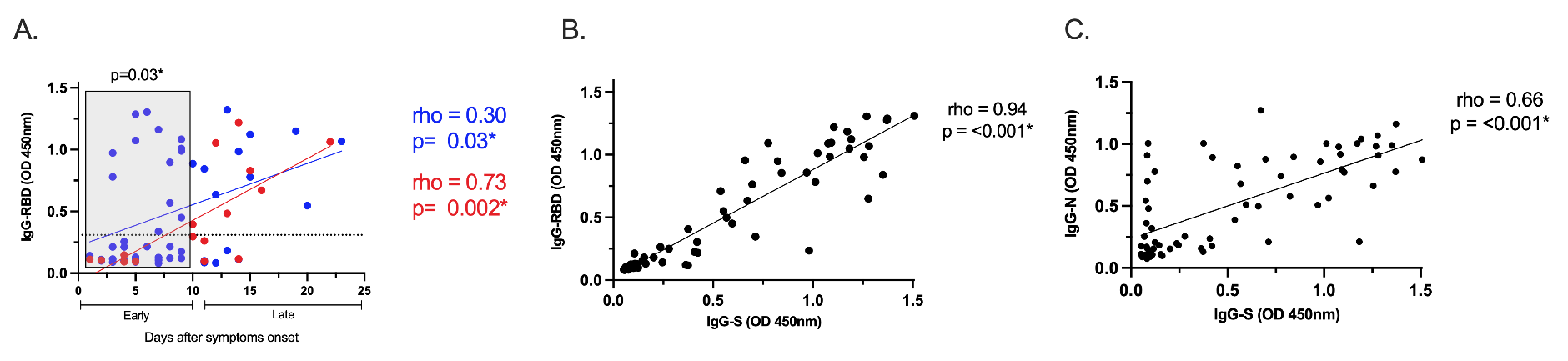
**

**Supplementary Figure 4. Delayed IgG-RBD response is associated with COVID-19-related mortality.** Correlation and linear regression analyses of the virus-specific responses IgG for RBD protein **(A)** with days after symptom onset in survivors and non-survivors of COVID-19**.** The Spearman test rho and p values are color-coded (blue for survivors, red for non-survivors). The dotted lines indicate the cutoff value. COVID-19 patients were classified into early (<10 days) or late (>10 days) phases post-symptom onset. The relative levels of the RBD-specific antibody responses during the early phase, marked in gray boxes (A), were compared between survivors and non-survivors. P values from the Mann–Whitney test are shown above each gray box. Correlation and linear regression analyses between IgG-S response with IgG-RBD response **(B)** and IgG-N response **(C)**. P <0.05* was considered significant across all analyses.

**Supplementary Figure 5.**

**
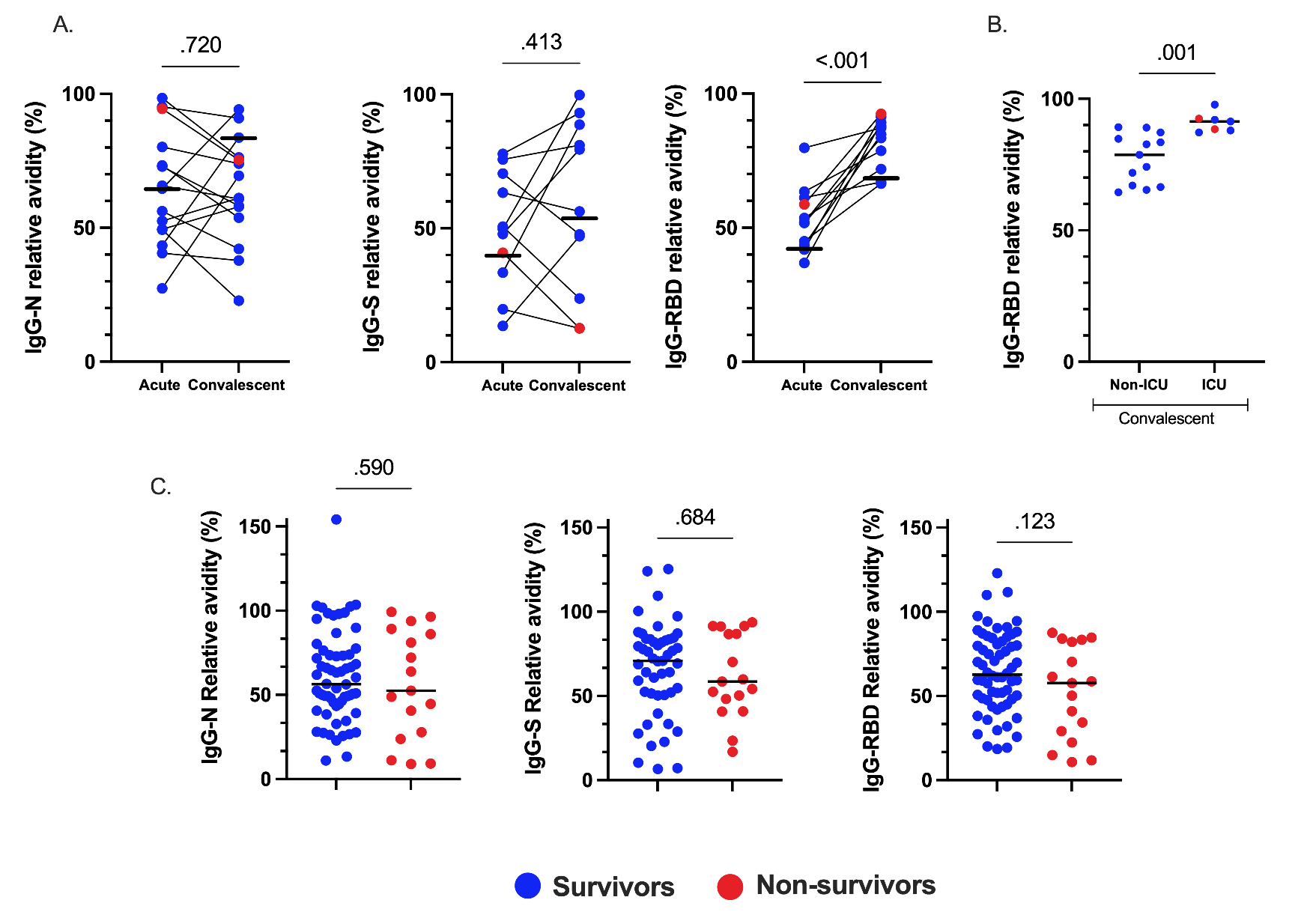
**

**Supplementary Figure 5. SARS-CoV-2-specific IgG avidity maturation is driven by RBD and is associated with COVID-19 severity.** Relative avidity of virus-specific IgG for N (left), S (center), and RBD (right) in acute and convalescent samples **(A)**. P values from the Wilcoxon test are shown. Relative avidity of IgG for RBD in convalescent samples from individuals who required ICU (intensive care unit) or who did not **(B)**. Relative avidity of virus-specific IgG for N (left), S (center), and RBD (right) in acute samples from survivors and non-survivors of COVID-19 **(C)**. Dots are color-coded (blue for survivors, red for non-survivors). P values from the Mann–Whitney test are shown. p <0.05 was considered significant for all analyses.

Supplementary Table 1. Age and comorbidities of the study population.

|  | Hospitalized COVID-19 | |  |
| --- | --- | --- | --- |
|  | Survivors | Non-survivors |  |
| Parameter | n=62 | n=17 | p-value |
| Range of age (years), n (%) |  |  |  |
| 18-29 | 10 (16) | 0 | 0.1^a^ |
| 30-39 | 12 (19) | 1 (6) | 0.27^a^ |
| 40-49 | 12 (19) | 1 (6) | 0.27^a^ |
| 50-59 | 8 (13) | 4 (24) | 0.28^a^ |
| >60 | 20 (33) | 11 (64) | 0.024^a^ |
| Hypertension, n (%) | 14 (22) | 6 (35) | 0.30^a^ |
| Type 2 diabetes, n (%) | 10 (16) | 3 (17) | 0.73^a^ |
| Obesity, n (%) | 31 (50) | 12 (70) | 0.17^a^ |

^a^ Fisher´s exact test, COVID-19, coronavirus disease-19; p <0.05 was considered significant.
